# A logical framework to study concept-learning biases in the presence of multiple explanations

**DOI:** 10.3758/s13428-021-01596-4

**Published:** 2021-06-18

**Authors:** Sergio Abriola, Pablo Tano, Sergio Romano, Santiago Figueira

**Affiliations:** 1 Universidad de Buenos Aires, Buenos Aires, Argentina; 2 ICC-CONICET, Buenos Aires, Argentina

**Keywords:** Concept learning, Learning biases, Propositional logic

## Abstract

When people seek to understand concepts from an incomplete set of examples and counterexamples, there is usually an exponentially large number of classification rules that can correctly classify the observed data, depending on which features of the examples are used to construct these rules. A mechanistic approximation of human concept-learning should help to explain how humans prefer some rules over others when there are many that can be used to correctly classify the observed data. Here, we exploit the tools of propositional logic to develop an experimental framework that controls the minimal rules that are *simultaneously* consistent with the presented examples. For example, our framework allows us to present participants with concepts consistent with a disjunction *and also* with a conjunction, depending on which features are used to build the rule. Similarly, it allows us to present concepts that are simultaneously consistent with two or more rules of different complexity and using different features. Importantly, our framework fully controls which minimal rules compete to explain the examples and is able to recover the features used by the participant to build the classification rule, without relying on supplementary attention-tracking mechanisms (e.g. eye-tracking). We exploit our framework in an experiment with a sequence of such competitive trials, illustrating the emergence of various transfer effects that bias participants’ prior attention to specific sets of features during learning.

Concept acquisition is a key and widely studied aspect of human daily cognition (Cohen & Lefebvre, 2005; Ashby & Maddox, 2011). Many researchers have claimed that a coding system and a set of rules underlie some of our abilities to acquire concepts (Nosofsky et al., 1994b; Tenenbaum et al., 2011; Maddox & Ashby, 1993), and it has been observed that we seem to learn concepts of objects with more ease when there are ‘simpler’ rules that can explain those groupings (Shepard et al., 1961; Nosofsky et al., 1994a; Rehder & Hoffman, 2005; Lewandowsky, 2011; Feldman, 2000; Blair & Homa, 2003; Minda & Smith, 2001).

In the real-world, humans learn concept descriptions while simultaneously deciding on which features to attend (Schyns et al., 1998); and the selected set of features usually determines the structure and complexity of the minimal rules that can describe the concept. For example, the concept *dog* can be explained as *a four-legged pet that is not a cat* or as *an animal for hunting, herding, pulling sledges or company*. Both descriptions are fully compatible with the concept *dog*, but our experience induces us to choose different relevant features to define the concept. While the first description of *dog* could be very well be given by a child having a dog at home, the second could be given by a shepherd or perhaps an ethologist. It is likely that the features used to describe *dog* by each agent allows them to compactly describe the concept, while simultaneously separating it from other concepts frequently encountered in their environment. Here, we ask about which features participants use to describe concepts, depending on the logical structure of the description using those features and also on their exposure to previous concepts. Why will someone use *cat* or *hunting* to define *dog*?

In propositional concept-learning experiments, participants are presented with a set of *examples*, each conformed of *N* propositional *features*, which can take positive or negative values. For instance, for *N* = 4 one example can be logically represented as the element (1,1,0,1), which takes positive values for the first, second and fourth features and negative for the second one, as illustrated in Fig. 1. A *concept* can be intuitively understood as a set of examples, some of them marked as belonging to the concept and the rest marked as not belonging, i.e. positive and negative examples. In Fig. 1 we show an example of an *underdetermined* concept, in the sense that, since the entire universe of examples is not shown (i.e. the 2^4^ possibilities), different determined concepts can be consistent with this smaller set when extending the set of examples to the full universe.

**Fig. 1:**
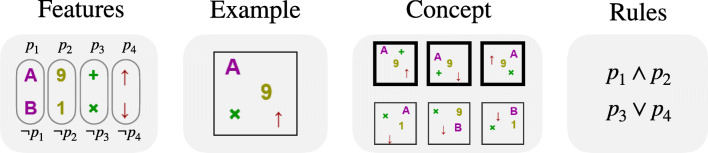
Illustration of the features {*p*_1_,*p*_2_,*p*_3_,*p*_4_}, the example (1,1,0,1), and a concept (positive example are marked with bold boundaries and negative examples with thin boundaries). The concept can be explained with the two minimal rules *p*_1_ ∧ *p*_2_ or *p*_3_ ∨ *p*_4_, depending on which features are used to build the rule (the first two features or the last two features, respectively)

A *rule* consistent with the concept is a logical formula built with the features and the conjunction (∧), disjunction (∨), and negation ($\lnot $) operators, which evaluates to true for objects belonging to the concept and false otherwise (e.g. *p*_1_ ∧ *p*_2_, where *p*_*i*_ is the *i*^*th*^ feature, see Fig. 1). The *minimal description length* (*MDL*) of a concept is the length of the shortest rule consistent with the concept (Grünwald & Grunwald, 2007) (here, the *length* of a formula is defined as the number of positive or negative occurrences of propositional symbols plus the number of occurrences of operators ∧ or ∨ contained in it; for example, the length of $p_{1} \land \lnot p_{3}$ is 3, and the length of $(p_{1} \land \lnot p_{3})\lor p_{2}$ is 5). Importantly, most studies of subjective difficulty with concept-learning are designed such that a *single* minimal rule can be used to describe the concept (e.g. *p*_1_ ∧ *p*_2_) (Ashby & Maddox, 2005; Feldman, 2000), even when the difficulty of finding the features that compose that rule (*p*_1_ and *p*_2_) is measured with attention-tracking mechanisms (e.g. Blair et al., 2009; Hoffman & Rehder, 2010). This limitation is possibly due to the prohibitively large number of rules that can be built with a given set of features, making it difficult to control which rules the participant might use when observing a set of examples. For instance, in order to determine the difficulty that participants have in learning the logical rule *p*_1_ ∨ *p*_2_, it is crucial to control that no other rule of reasonable complexity can explain the concept (e.g. *p*_1_ ∧ *p*_3_). In this work, we use the tools of propositional logic to build an experimental framework that allows us to present examples consistent with two (or more) chosen rules, depending on which features are observed. For instance, the concept shown in Fig. 1 is consistent with the explanation *p*_1_ ∧ *p*_2_ *and also* with the explanation *p*_3_ ∨ *p*_4_, depending on which features are observed. In general, the experimenter can choose any pair of rules that use any number of (non-overlapping) features, and our framework guarantees that the presented examples are only consistent with the two minimal rules chosen by the experimenter. Then, by presenting novel examples that are consistent with only one of the previous rules, the experimenter can determine which rule the participants internally used to learn the concept, and thus which features they attended to.

Presenting rules *A* and *B* (e.g. *p*_1_ ∧ *p*_2_ and *p*_3_ ∨ *p*_4_) using the same set of examples has several experimental advantages over separately presenting a set of examples consistent with rule *A* and then a set of examples consistent with rule *B*. Some of the advantages are:

1. When comparing the relative difficulty of learning *A* and *B* in the same participant, presenting the examples separately makes it hard to overcome transfer effects that cause subjective difficulty to depend on the history of concepts learnt previously in the task, and cause different relative difficulties if *A* is learnt before *B* compared to *B* being learnt before *A* (see for example Tano et al., 2020). The experimenter could compare learning times for *A* and *B* across participants, but for reasonably hard rules there are very large idiosyncratic differences in learning difficulties which greatly increases the variance of learning times (see for example Feldman, 2000), and also the experimenter cannot normalize the past history of each participant before the experiment. On the other hand, presenting *A* and *B* simultaneously via the same set of examples allows us to directly measure which of the two rules is most easily found by the participant, when the two are presented under exactly the same experimental conditions.
2. The fact that rule *A* is learnt more easily than *B* when presented separately does not necessarily mean that the same happens when presented jointly. This could not hold if there is an interaction between the logical operators being learnt (that compose the rules *A* and *B*) and the search mechanism used to find the corresponding rules. For instance, the search mechanism that allows humans to find a disjunction rule consistent with the examples could interact with the mechanism that allows to find conjunctions, an interaction that could only be characterized when the conjunction and disjunction are presented at the same time.
3. Our framework allows us to test second-order subjective difficulty effects (e.g. rule *A* is learnt faster if presented jointly with rule *B* than with rule *C*), as well as second-order transfer learning effects (e.g. participants learn more rapidly rule *C* if they have first observed rule *A* jointly presented with an arbitrary rule *B*_1_, compared to *A* coupled with a different rule *B*_2_).
4. If one is interested in which features are preferentially observed by the participant in a given trial (e.g. features {*p*_1_,*p*_2_} or {*p*_3_,*p*_4_}), one could simply choose the same logical structure for *A* and *B* (e.g. making *A* and *B* equal to *p*_1_ ∧ *p*_2_ and *p*_3_ ∧ *p*_4_) and test whether *A* or *B* is learnt by the participant. Then, any preference for learning *A* over *B* could only be due to a preference over the features themselves ({*p*_1_,*p*_2_}), and not for the logical description of the concept using those features (this is, ***⋅***∧***⋅***).

We illustrate these advantages in an experiment in which participants are presented with a sequence of 6 trials, observing in each trial a set of examples consistent with two alternative rules. We illustrate advantage (1) and (2) discussed above by presenting a conjunction together with a disjunction; and a simple rule together with a complex rule. Then, we show that after observing in several trials that a subset of features is useful to find concise rules, we induce in the participants a bias to preferentially describe concepts using those features; this bias was tested exploiting advantage (4).

## Experiment

### Participants

The experiment was conducted as a Human Intelligence Task (HIT) in Amazon’s Mechanical Turk (Crump et al., 2013; Buhrmester et al., 2011; Stewart et al., 2015). There were 100 participants, self-selected workers that saw, accepted, and finished the published HIT. We required workers to have a HIT approval rate of 95*%* or more. Workers were informed that the payment for completing the experiment was going to be of 1.5 US dollars, and that 1 out of 20 participants would be randomly assigned a bonus of 10 dollars, regardless of their performance in the experiment’s tasks as long as they finished the experiment (but note that trials did not end until they correctly learned each concept).

For exclusion criteria, see the Appendix ??.

### Experiment setup

**Fig. 2:**
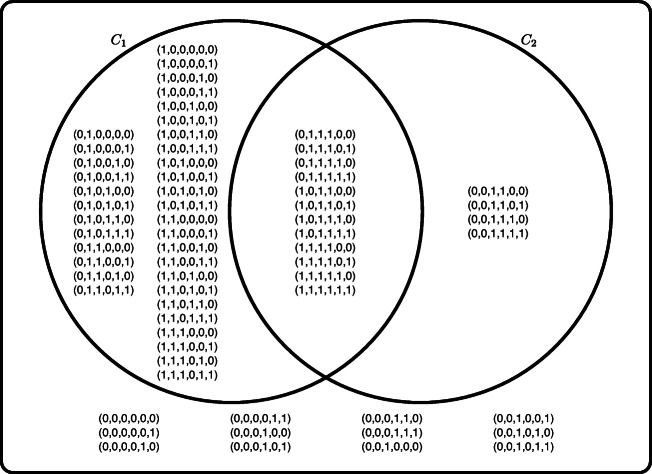
An example of a pair of concepts *C*_1_ and *C*_2_ with 6 features. Concept *C*_1_ can be described by *φ*_1_ = *p*_1_ ∨ *p*_2_, and *C*_2_ by *φ*_2_ = *p*_3_ ∧ *p*_4_. This is just a schematic illustration of where each element (tuple) is placed with respect to concepts. These concepts correspond to the ones used in Trial 1 of the actual experiment. However, elements in the actual experiment are not represented in this way (i.e. as tuples of zeroes and ones)

The main idea of our experimental framework is schematized in Fig. 2. The participants observe an *underdetermined* concept. This concept is presented to the participants as a set of elements that belong to it (positive examples), and a set of elements that do not (negative examples). In Fig. 2, the elements marked as positive examples are the ones in the intersection of the two concepts and the negative examples are the ones outside of both concepts. Importantly, the listing is incomplete, in the sense that not all elements of the universe are shown. The critical insight is that, when extending the set of examples to the full universe, there is more than one possible concept that is consistent with the observed examples. For example, in Fig. 2, the presented examples are consistent with the minimal rule of *C*_1_ (i.e. *φ*_1_ = *p*_1_ ∨ *p*_2_) *and also* with the minimal rule of *C*_2_ (i.e. *φ*_2_ = *p*_3_ ∧ *p*_4_). As we explain in the rest of this section, choosing *C*_1_ and *C*_2_ appropriately can be exploited to control the minimal rules that are consistent with the examples that participants observe.

**Fig. 3:**
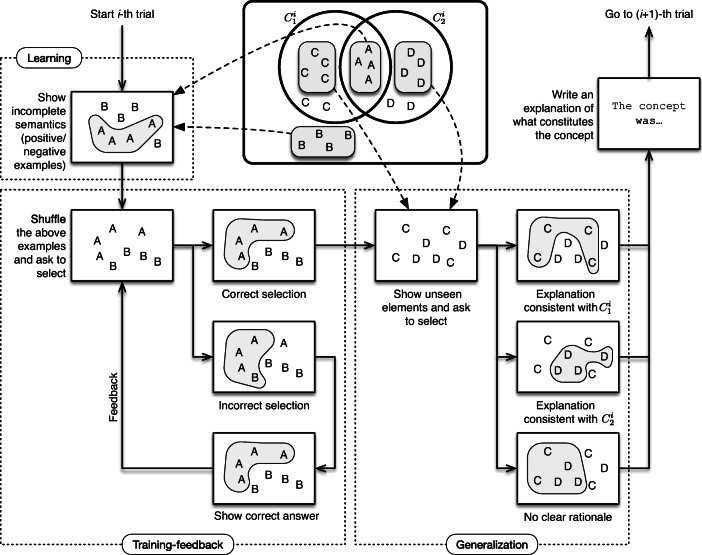
The scheme of our experimental framework for studying concept learning in the presence of multiple explanations. We illustrate the three phases that constitute each trial: learning phase, training-feedback phase and generalization phase. Elements are represented with letters A, B, C and D (for example, the four letters A in the intersection represent four different elements in the intersection). The depicted number of such letters A, B, C or D is irrelevant (for example, there would be 12 A s and 4 D s for concepts of Fig. 2)

The actual experiment that we implemented consists of a sequence of 6 trials constructed in this manner. We now expand the 3 stages that compose each *i*-th trial of the experiment. For a better understanding, see Fig. 3, which consists of a schematic view of one trial. Note that this figure is merely illustrative and does not aim to describe the details of a trial, but rather the sequence of phases and the logical flow within a trial. In particular, note that the number of elements A’s, B’s, C’s and D’s in the figure are not meaningful, as they vary from trial to trial along the experiment. The actual concepts used in each trial, as well as the number of positive and negative examples is listed in Table 1 (groups X,Y are only relevant for Hypothesis III, so they can be ignored for now), and more details of the actual implementation can be found in “Representational details” and “Details of the experiment’s structure”.

1. **Learning stage.** The participant is exposed to a set of ‘in’ elements corresponding to ${C^{i}_{1}}\cap {C^{i}_{2}}$ (marked as ‘A’ in Fig. 3), and a set of ‘out’ elements corresponding to the *complement* of ${C^{i}_{1}}\cup {C^{i}_{2}}$ (marked as ‘B’ in Fig. 3).
  We call these shown elements ‘positive examples’ and ‘negative examples’, respectively. Note that this information is incomplete, in the sense that not all possible examples are shown to the participant (as the only examples that are shown from ${C^{i}_{1}}\cup {C^{i}_{2}}$ are those in ${C^{i}_{1}}\cap {C^{i}_{2}}$). In the illustrative example of Fig. 2 (corresponding to concepts of Trial 1 of the actual experiment), 24 elements would be shown: the 12 positive examples in the intersection of *C*_1_ and *C*_2_, and the 12 negative examples outside of both *C*_1_ and *C*_2_. The participant is asked to learn the concept represented by positive examples.
  As we prove formally in Appendix A, the experimental design guarantees that there are only two propositional rules (*φ*_1_ and *φ*_2_ in Fig. 2), minimal over their respective sets of features, such that: *(1)* they are *consistent* explanations for shown examples (this is, they satisfy positive examples but do not satisfy negative examples), *(2)* they use different features from each other (e.g. {*p*_1_,*p*_2_} in *φ*_1_ and {*p*_3_,*p*_4_} in *φ*_2_) and, importantly, *(3)* *any* rule consistent with the examples must use a superset of the set of features of at least one of these minimal rules. For instance, in Fig. 2 any rule that only uses {*p*_2_,*p*_3_} cannot explain the examples, since (1,**0**,**1**,1,1,1) is a positive example but (0,**0**,**1**,0,1,1) is a negative example. Any rule that can consistently explain the examples must mention a superset of {*p*_1_,*p*_2_} (e.g. {*p*_1_,*p*_2_,*p*_3_}) or a superset of {*p*_3_,*p*_4_}. The proof of this condition is shown in Theorem 3, but we also sketch it here. Observe that in Fig. 2 the negative example (0,**0**,**1**,0,1,1) was constructed from the positive example (1,**0**,**1**,1,1,1) by flipping the values of *p*_1_ and *p*_4_, and doing so results in an element that is inconsistent with both *φ*_1_ and *φ*_2_. When an alternative explanation leaves unused some features *p*,*q* that appear in *φ*_1_ and *φ*_2_ respectively, there must be some element that satisfies both rules *φ*_1_,*φ*_2_, but none of them is satisfied when the values of *p* and *q* are flipped. Since the truth value of the alternative rule is maintained when features that do not appear in it change, and since we are showing as positive examples all elements that satisfy both rules *φ*_1_,*φ*_2_ and as negative examples all those that satisfy none of them, such alternative explanation must be inconsistent with the shown data.
  These three conditions guarantee that the experimental procedure illustrated in Fig. 2 is a logically sound method to present a concept consistent with two minimal rules chosen by the experimenter (*φ*_1_ and *φ*_2_), depending on which features the participant use to build the rule.
2. **Training-feedback stage.** The *same* examples of the learning stage are shown to the participant, but this time without indicating whether they are negative or positive and in a shuffled order. The participant is asked to tag each element as ‘in’ or ‘out’, in the same way they were tagged in the previous step. If all elements are classified correctly, the participant proceeds to the next stage. Otherwise, the participant is informed about the mistakes in their tagging, and after that the training-feedback stage starts again.
3. **Generalization stage.** *Previously unseen* elements are shown to the participant^1^. These elements are taken from ${C^{i}_{1}}\setminus {C^{i}_{2}}$ and from ${C^{i}_{2}}\setminus {C^{i}_{1}}$ (here, ‘∖’ denotes set difference). These elements are respectively marked as ‘C’ and ‘D’ in the scheme of Fig. 3. The participant is asked to identify those elements that correspond to the concept learnt in the learning stage. After they do so, the next trial starts. If the participant selects those in ${C^{i}_{1}}\setminus {C^{i}_{2}}$, the concept learnt in the Learning stage was ${C^{i}_{1}}$, and if the participant selects those in ${C^{i}_{2}}\setminus {C^{i}_{1}}$, the concept they learned was ${C^{i}_{2}}$. Continuing with the example from Fig. 2, this process would allow us to determine if the participant was thinking in a rule with the features {*p*_1_,*p*_2_} (namely, *φ*_1_) or {*p*_3_,*p*_4_} (namely, *φ*_2_) to explain the concept. Of course, in practice the participant can select other elements, with no clear rationale.
  Once the participant chooses the elements, they are asked to write an explanation of what constitutes the concept; this answer is not part of the data analysis, except that it allows us to exclude participants that are using methods outside the scope of the experiment (such as taking pictures). Additionally, the written answers serve as an extra sanity check of whether the participants are actually thinking in a way consistent with the framework of propositional logic (see Appendix ?? for observations on the written explanations obtained in the experiment).

**Table 1:** The trials of the experiment

Trial	Groups	${{\varphi ^{i}_{1}}}$	${{\varphi ^{i}_{2}}}$	Shown features	Tested hypotheses				#Positive (#Negative) examples shown
					I	II	III	IV	
*i* = 1	X, Y	*p*_1_ ∨ *p*_2_	*p*_3_ ∧ *p*_4_	*p*_1_ to *p*_6_	∙			∙	12 (12)
*i* = 2	X, Y	$\lnot p_{1} \land p_{2}$	$p_{3} \lor \lnot p_{4}$					∙	12 (12)
*i* = 3	X	*p*_1_ ∧ *p*_2_	MDL 15				∙		10 (18)
	Y	*p*_5_ ∧ *p*_6_	MDL 15						
*i* = 4	X, Y	$ \lnot p_{5} \land p_{6}$	MDL 15				∙		10 (18)
*i* = 5	X, Y	*p*_7_ ∧ *p*_8_	MDL 15	*p*_3_ to *p*_8_		∙			10 (18)
*i* = 6	X, Y	$\lnot p_{7} \land \lnot p_{8}$	*p*_3_ ∧ *p*_4_			∙			4 (36)

IHere ${\varphi ^{i}_{1}}$ and ${\varphi ^{i}_{2}}$ represent the two competing concepts ${C^{i}_{1}}$ and ${C^{i}_{2}}$ at the *i*-th trial (we denote each concept by the shortest propositional rule whose semantics describes the concept). By “MDL15” we denote a concept whose shortest rule is of length 15 (and made of three propositional symbols other than the competing rule in the corresponding trial, see “MDL bias” for details). In all trials the full universe size is 2^6^ = 64, corresponding to all possible elements over 6 propositional features. We indicate how participants were divided into groups X and Y, which was used only for Hypothesis III. We also indicate which features were shown in the examples, which hypothesis where tested, and the number of positive and negative examples shown in learning and training phases for each trial

More details of the experiment and its structure can be found in “Methodology”, particularly in “Representational details” and “Details of the experiment’s structure”.

### Experiment trials

The set of trials chosen in the experiment (Table 1) aims to reveal the biases that cause participants to choose one set of features over another in this framework where both sets of features have their own minimal rules consistent with the observed positive and negative examples. For instance, in Fig. 2, what causes participants to choose {*p*_1_,*p*_2_} versus {*p*_3_,*p*_4_} to explain the concept? Our hypothesis is that a key inductive bias is simply the frequency with which a subset of features was used previously to explain past concepts. We name this bias as *feature stickiness*.

We now present the main hypotheses of this work, and their relation with the various experimental trials.

#### **Hypothesis I**

In Trial 1 we explore whether the same factors that determine rule-learning difficulty when learned in isolation also determine which features participants use when explaining a set of examples consistent with two minimal rules. Particularly, it is well known that concepts involving logical conjunctions are learned faster than concepts involving logical disjunctions (Bourne, 1970).

In Trial 1, the minimal consistent rule is a disjunction if the observed features are {*p*_1_,*p*_2_}, and a conjunction if the observed features are {*p*_3_,*p*_4_}. Importantly, unlike in other concept-learning experiments, both the two-feature disjunction and conjunction are consistent with the observed set of examples. We hypothesize that the learning bias that causes the conjunction to be learnt more easily than the disjunction will also carry over to this framework were both explanations are possible (using different features). As explained before, we use the generalization stage of Trial 1 to determine if participants understood the concept using {*p*_1_,*p*_2_} (corresponding to a disjunction) or using {*p*_3_,*p*_4_} (corresponding to a conjunction).

This hypothesis was preregistered as:

“In a scenario of two possible explanations for a concept, one of which can be modeled by the logical ∧ between two features and other which can be modeled by the ∨ between two other features, most people will find the ∧ explanation over the ∨ explanation.”

#### **Hypothesis II**

The *feature stickiness* bias is tested in Trials 5 and 6 of the experiment. After participants have gained sufficient experience with the task, in Trial 5 participants encounter a set of examples consistent with two minimal explanations, a very simple one that uses features {*p*_7_,*p*_8_} and a very complex one that uses {*p*_4_,*p*_5_,*p*_6_}. This leads participants to explain the concept using {*p*_7_,*p*_8_}, or otherwise they would have to discover an excessively complex explanation. Therefore, we hypothesize that in this case most participants would select the features {*p*_7_,*p*_8_}^2^.

In the following concept (Trial 6), participants must choose between explanations that use the previously useful features {*p*_7_,*p*_8_}, or another fresh set of features {*p*_3_,*p*_4_}. We hypothesize that participants are more likely to explain the concept using {*p*_7_,*p*_8_}, only because these features were useful in the previous concept. Also, recall that explanations that use a set of features containing either {*p*_7_,*p*_8_} or {*p*_3_,*p*_4_} are also compatible. For example, in Trial 6 the explanation $p_{3} \land p_{4} \land \lnot p_{7}$ is compatible with the observed examples. We are also interested in these rules (e.g. we think it is more likely that participants will use {*p*_7_,*p*_8_,*p*_3_} than {*p*_3_,*p*_4_,*p*_7_}). The seven elements chosen for the generalization stage of Trial 6 allows us to do precisely this: 7 elements appear on the screen, with *p*_3_,*p*_4_,*p*_7_,*p*_8_ respectively equal to (1,1,1,1), (1,1,0,1), (1,1,1,0), (1,1,0,0), (1,0,0,0), (0,1,0,0), (0,0,0,0). These elements are respectively consistent with the minimal rules *p*_3_ ∧ *p*_4_, $p_{3} \land p_{4} \land \lnot p_{7}$, $p_{3} \land p_{4} \land \lnot p_{7} \land \lnot p_{8}$, $p_{3} \land \lnot p_{7} \land \lnot p_{8}$, $p_{4} \land \lnot p_{7} \land \lnot p_{8}$ and $\lnot p_{7} \land \lnot p_{8}$. Importantly, none of the elements is consistent with more than one of the two minimal rules.

This hypothesis was preregistered as:

If a person has used a set of features in the construction of an explanation for a concept, it is more likely that she will also find an explanation containing those features in the following trial.

#### **Hypothesis III**

We address the question of whether the feature stickiness bias represents a computational advantage in itself. More concretely, we ask if participants find a consistent rule *faster* when they are reusing the same features as in the previous trial. Note that this is a distinct phenomenon from Hypothesis II, which is concerned with preferential selection and not with times. We test this question, independently of the effect of the feature stickiness bias, in Trials 3 and 4 of the experiment. In Trial 3, we separate participants into groups X and Y. In the same manner as in Trial 5, in Trial 3 group X is biased to learn the rule using {*p*_1_,*p*_2_}, and group Y using {*p*_5_,*p*_6_}. In the next trial (Trial 4), participants are biased to learn the rule using {*p*_5_,*p*_6_}. We hypothesize that participants from group Y will learn concept ${C^{4}_{1}}$ faster than participants from group X, given that they are reusing the same features they used in the previous trial.

This hypothesis was preregistered as:

When a concept can only be reasonably described by a given set of features, a person will find this description faster if that same set of features was useful for her in the immediately previous trial.

#### **Hypothesis IV**

Another question, tested with Trials 1 and 2, examines the relative strength of feature bias versus operator bias. That is, we want to determine whether there is some strong effect that clearly biases attention towards features (or rather toward operators) that have previously been found useful for describing concepts. We test this by switching the operator (∨/∧) that each pair of features can use to form a useful rule in each trial, and by then comparing the number of participants that explain the shown examples of Trial 2 by reusing the same features from Trial 1 versus those that reused the operator but used different features.

This hypothesis was preregistered as:

In a scenario where both features and operators are repeated from a trial to the next, there will be a stickiness effect favoring one of them over the other.

## Methodology

### Preregistration and data

This study’s methodology, data collection procedures, sample size, exclusion criteria, and hypotheses were preregistered on the Open Science Framework (OSF) in advance of the data collection and analysis. The preregistration can be accessed at https://osf.io/mgex3, while the obtained data and the experiment played by the participants is available at https://osf.io/gtuwp/.

In this work we also make some exploratory (not preregistered) analyses: we correct for verbal explanations that are not consistent with a positive interpretation of the concept for Hypothesis I, we exclude outliers from the analysis in Hypothesis II, and we consider the effect of the participant’s learning history beyond the immediately previous trial in Hypothesis II. We also explicitly analyse, in this framework of multiple consistent explanations, the difference in revealed difficulty between rules of greatly differing minimal length.

### Representational details

The underlying mathematical structure of the trials uses propositional variables, valuations, and sets of valuations. However, these are not shown abstractly, but rather are represented via correspondences to features (symbols), elements (boxes), and concepts (collections of elements).

We next describe details of the representations used for the experiment and its competing concepts.

#### Features—propositional variables

The experiment encompasses eight propositional variables: $p_{1},\dots ,p_{8}$. Each variable can take one of two possible values, and these values are graphically represented by icons. For instance, *p*_1_ can be assigned icon ‘A’ or icon ‘B’, representing the values 1 (positive) and 0 (negative) respectively, *p*_3_ can be assigned a ‘ + ’ icon or ‘×’ icon representing 1 and 0 respectively, and so on.

**Fig. 4:**
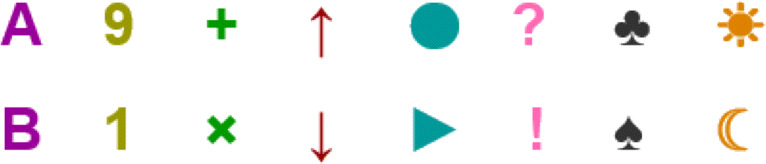
Pictured above are the features, the visual representation of the positive and negative values of the propositional variables. The upper row represents positive values of the propositional variables, while the lower row represents their negation

Figure 4 shows the pairs of values for each of the eight propositional variables. The assignment of pairs of icons to propositional variables is randomized at the start of the experiment, and does not vary within the experiment. The reason to choose icons instead of (colored) values 0,1 is to avoid the possibility of mentally learning a concept using ‘counting’ or other operators not present in propositional logic. For example, showing explicit {0,1} values, a possible explanation for a concept could be *more than 3 ones*, but such a description would be much harder in the icon-based representation, since different propositional variables have no symbols in common. In “Notes on the experiment design” we discuss more details on these considerations.

#### Elements (boxes)—valuations

**Fig. 5:**
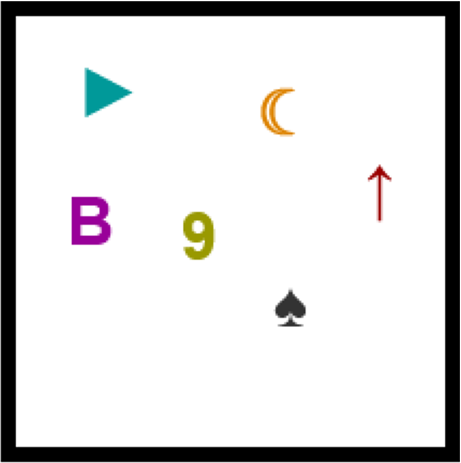
An element. This box containing features is the visual representation of a valuation over six propositional variables. Here the box appears with a neutral border, but boxes in the experiment always appear with a border that denotes whether they are positive or negative examples. The position of the symbols is irrelevant for the concepts, and is randomly assigned

A valuation over the propositional variables is visually represented as a square/box with the values (icons) of all propositional variables set at random positions inside the square. We call such representation an ‘element’ (see Fig. 5 for an example of such an element). The reason for choosing this representation is to avoid directional biases that could influence learning, and to exclude ordering and other operators from the language of thought (see “Notes on the experiment design” for more details). Each time an element is shown (in particular, within the loop in the training-feedback) a new random position is chosen for the propositional features inside it.

#### Undetermined concepts—sets of positive/negative valuations

**Fig. 6:**
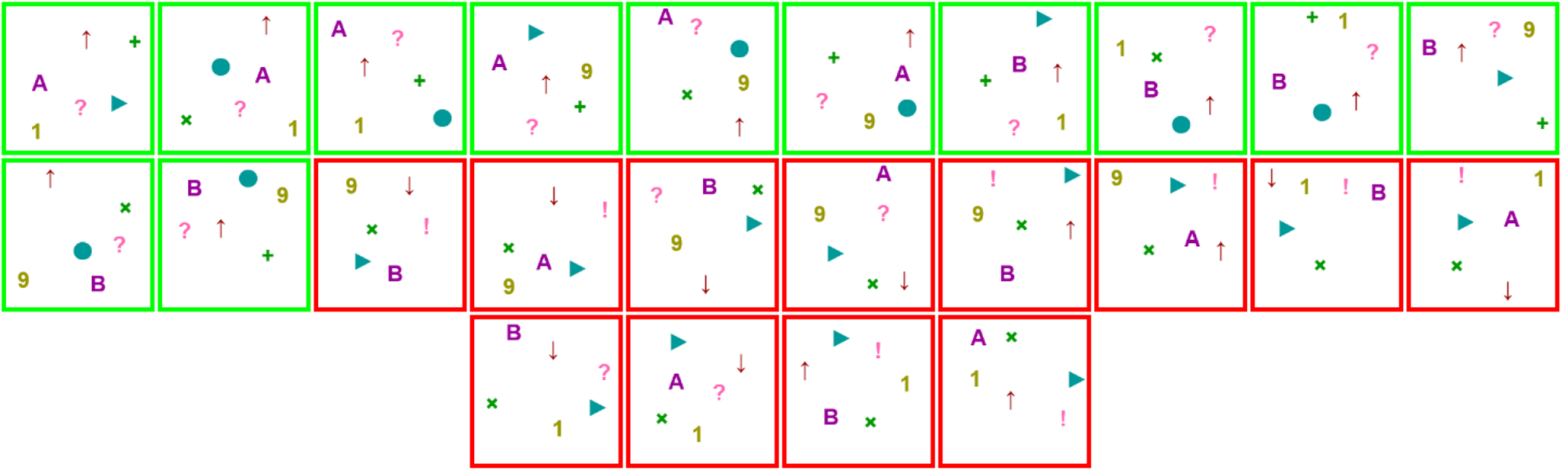
A sequence of positive and negative examples in a learning stage, corresponding to Trial 1. A green border informs the participant that the element belongs to the concept, while a red-bordered one informs that it does not belong to the concept. In this case, the examples could be explained as either ‘boxes containing both an upwards pointing arrow and a question mark’ or as ‘boxes that contain a circle or a plus sign’, but note that these two rules determine different concepts over the complete set of possible elements

The concept shown in the learning stage of a trial corresponds to two non-overlapping sets of valuations, and these two sets do not cover all possible valuations. This is represented as a sequence of ‘in’ and ‘out’ elements, with no information given on elements that are not shown. At the learning stage, shown ‘in’ elements (positive examples) are represented as a green box and shown ‘out’ elements (negative examples) as a red box. See Fig. 6 for an example of a tagged sequence of elements used in the learning stage. Each time the concept is presented, we shuffle the order in which their positive and negative examples are shown, but always presenting all positive examples first (also, each valuation is assigned new random positions for the features inside the corresponding box).

#### (Hidden) concepts—formulas

Over the full set of valuations, a concept is simply the set of valuations that positively describe it. The two hidden concepts for each trial correspond to the valid and minimal generalizations that can be made from the incomplete concepts. They can be described as the semantics of the two propositional formulas (rules) that can be used to explain the incomplete concept (see Table 1); while these rules coincide over the incomplete universe shown in the learning stage, they differ over the set of all valuations. For more details, recall the beginning of “Experiment setup” and its Item 1. For technical details, see Appendix A.

In Table 2 we summarize the main logical terminology used to define formal semantics, and its representational counterpart adopted in our experimental setup.

**Table 2:** Terminology used for explaining the formal semantics of Boolean logic both in mathematical terms and in the representational terms used in the experiment

Mathematical terminology	Representational terminology
**Valuation**: a tuple $\overline v=(v_{1},\dots ,v_{n})$ where each *v*_*i*_ is 0 or 1.	**Element**: a square with *n* symbols inside (see Fig. 5). There is an implicit coding shown in Fig. 4 (for example, *v*_1_ = 1 is represented by a ‘A’ and *v*_1_ = 0 is represented by an ‘B’, *v*_3_ = 1 is represented by a ‘ + ’ and *v*_3_ = 0 is represented by a ‘×’, and so on).
**Propositional variable**: *p*_*i*_ takes value *v*_*i*_ under valuation $\overline v=(v_{1},\dots ,v_{n}).$	**Feature**: *p*_*i*_ is represented, via the implicit coding, by one of the pairs of Fig. 4 within an element representing $\overline v$.
**Concept**: a set *U* of valuations representing the ‘positive’ ones (for example, *C*_1_ in Fig. 2). Notice that the negative valuations are just all valuations not in *U*.	**Concept**: any categorization that divides the space of all possible elements in either positive (all those elements that belong to *U*) or negative (elements that do not belong to *U*).
Observe that any concept *U* has a corresponding minimal **formula/rule** *φ*_*U*_ that characterizes it (i.e. *φ*_*U*_ is true over the valuations in *U*, and is false over the complement of *U*).	
**Undetermined concept**: a pair 〈*U*,*V* 〉 of sets of valuations representing the ‘positive’ and ‘negative’ ones respectively such that *U* ∩ *V* = *∅* and *U* ∪ *V* is not the set of all valuations (for example, the pair $\langle C_{1}\cap C_{2}, \overline {C_{1}\cup C_{2}}\rangle $ in Fig. 2).	**Undetermined concept**: a sequence of positive elements (green border) representing *U*, and negative elements (red border) representing *V* (see Fig. 6 for an example). Importantly, *U* and *V* do not cover the full universe of possibilities spanned by the features.
Observe that an undetermined concept 〈*U*,*V* 〉 can be generalized in more than one way by (minimal) formulas *φ*_1_ and *φ*_2_ such that *a)* *φ*_*i*_ (*i* = 1, 2) is true over all valuations in *U*, and false over all valuations on *V*, and *b)* the set *all* of positive valuations where *φ*_1_ is true is different from the set of *all* valuations where *φ*_2_ is true. For example, the undetermined concept shown in each trial *i* of the experiment can be generalized via the two corresponding minimal formulas ${\varphi ^{i}_{1}}$ and ${\varphi ^{i}_{2}}$ shown in Table 1.	

### Details of the experiment’s structure

As we explain in “Experiment”, each instance of the experiment consists of 6 trials where the participants must learn a concept from an incomplete universe. The presented positive and negative examples are such that there are exactly two minimal rules (up to logical equivalence) in propositional logic that *1)* are consistent explanations for the shown examples; *2)* use disjoint sets of variables from each another; and *3)* any rule consistent with the examples must use a superset of the set of features of at least one of these minimal rules. This experimental setup will allow us to distinguish which of these rules best represents the way that the participant learned the concept. See Appendix A for technical details.

Observe that merely asking the participant to select already seen elements does not give us any obvious insight into the internal process that derived into the learning of the concept; even if they internalized the concept using one of the two rules, it would remain uncertain which one they used, as both rules have the same semantics over the shown universe. In order to distinguish between these two cases, we use a generalization stage where previously unseen elements of the universe are shown, and the participant must select those that they believe belong to the concept. Of these new elements, some are consistent with only one of the rules, and other are consistent only with the other rule^3^. Furthermore, immediately afterwards we ask for a written explanation of what characteristics the participant thinks describe the concept.

Structurally, the experiment begins with the (hidden) assignment of the participant to one of two groups X or Y (see Table 1) and the exposition to a page with instructions. Afterwards, there are 6 trials with the following structure: they begin with a learning stage; they continue to a training stage where they get feedback if they fail to correctly select the elements that belong to the concept; a generalization stage where they must choose between elements of the universe that were not shown previously; and, in all but the last trial, a stage where the participants can rest between trials.

In what follows, we describe each stage of the experiment plus the introductory page, with a greater detail than that of “Experiment setup”.

#### Introduction and explanation

This is the page that subjects are shown at the beginning of the experiment. It describes the main task they will be asked to perform: that of learning from examples to distinguish what kind of ‘boxes’ belong to a certain concept. These elements are represented as a collection of 6 symbols, no more than one from a same pair. It is also informed that the position of the symbols does not matter. See Fig. 5 for an example element.

When the subject indicates they have finished reading the instructions, they are sent to a fullscreen page with three multiple-choice questions whose purpose is to verify that the participant has understood the instructions; if they miss some answer, they are returned to the previous page and the cycle is repeated until they succeed.

If the participant answers correctly, they are now ready to begin, and the phases “The learning phase”, “The training–feedback phase”, and “The generalization phase” are then entered sequentially for each of the 6 trials.

#### The learning phase

In this phase of a Trial *i*, the participant is shown a set $S^{i} \subsetneq U^{i}$, a proper subset of elements from the current universe. Each universe syntactically corresponds to all the combinations of truth values for 6 propositional variables taken from the set {*p*_1_,*p*_2_,*p*_3_,*p*_4_,*p*_5_,*p*_6_,*p*_7_,*p*_8_}, thus spawning a set *U*^*i*^ of 64 elements. On the semantic side we call ‘features’ the visual representations of the propositional variables, and these representations remain fixed through the experiment (recall Fig. 4).

The elements of *S*^*i*^ are shown as boxes, some of which have green border (denoting a positive example, that the element belongs to the concept), while the rest have red borders (denoting a negative example, that they do not belong). The green-bordered boxes are shown first, with the red-bordered ones appearing after the last box with green border. See Fig. 6 for an example learning set.

If the graphical representations are abstracted away to the underlying basic structure, there are two propositional rules ${\varphi ^{i}_{1}}$ and ${\varphi ^{i}_{2}}$ (of minimum length in their class of logically equivalent rules, see Table 1) whose semantics correctly classify the positive and negative examples shown. If we call ${C^{i}_{1}}, {C^{i}_{2}}$ the sets of valuations that satisfy ${\varphi ^{i}_{1}}, {\varphi ^{i}_{2}}$, respectively, we have that $S^{i} = ({C^{i}_{1}} \cap {C^{i}_{2}}) \cup \overline {({C^{i}_{1}} \cup {C^{i}_{2}})}$. The rules ${\varphi ^{i}_{1}}, {\varphi ^{i}_{2}}$ use at most^4^ 3 of the 6 propositional variables available in *U*^*i*^, and the two rules do not have propositional variables in common.

When the participant believes they have learned which elements belong to the concept, they can click a button to proceed to the next stage.

#### The training–feedback phase

**Fig. 7:**
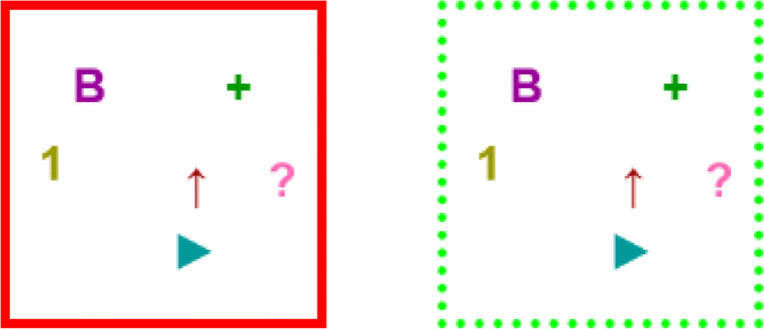
An unselected element, to the left, is represented by solid red borders. The same element in a selected state, to the right, is indicated by dotted green borders

In this phase, the participant is shown a random rearrangement of *S*^*i*^, with all the elements now surrounded by a red-bordered square. The subject must click exactly those elements (if any) they believe belong to the concept —changing them to a dotted green border (see Fig. 7)— and then has to click a button to submit their choice.

**Fig. 8:**
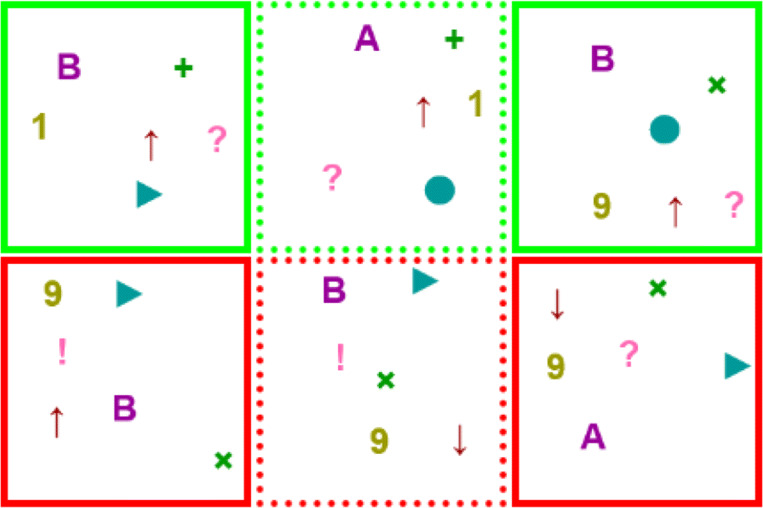
A partial section of the feedback resulting from a wrong selection. A solid green border means that the box was correctly selected as belonging to the concept. A solid red border means that it was correctly left unselected, meaning that it did not belong to the concept. A dotted green border means the box belongs to the concept but was not selected, and a dotted red border means that the box does not belong to the concept but was selected

If their selection is incorrect, the participant is shown which elements they misclassified (either by clicking them incorrectly or by failing to click them, see Fig. 8). When they click a button to continue, they restart this stage (with a fresh randomization).

When the participant finally makes the correct selection, they continue to the next phase.

#### The generalization phase

In this phase, the participant is shown a subset of *U*^*i*^∖*S*^*i*^ (namely, in $({C^{i}_{1}} \cup {C^{i}_{2}}) \backslash ({C^{i}_{1}} \cap {C^{i}_{2}})$), that is, a selection of elements that were *not* present in the learning phase (hence nor in the training phase). The participant must classify which of these elements they think belong to the concept. The participant does not receive feedback on the choices they make here. Except for the sixth trial, part of these elements satisfy the rule ${\varphi ^{i}_{1}} \land \lnot {\varphi ^{i}_{2}}$, while the rest satisfy ${\varphi ^{i}_{2}} \land \lnot {\varphi ^{i}_{1}}$. Thus —assuming the participant learned the concept via a process akin to a representation of one of the two rules—this phase crucially serves to distinguish which rule they have learned, if any.

After this selection, the participant is asked to submit a written explanation of what characteristics they think constitute the concept. This written explanation serves as an additional validation of whether they are thinking in a way describable by propositional logic according to our assumptions, or if rather they are using other methods (memorization, pen and paper, screenshots, other logics or formalisms, etc.).

### Notes on the experiment design

The elements, universes, and rules that constitute our experiment are devised in terms of propositional logic. However, it is important to be careful with the semantics, i.e. the way elements are actually shown to the participants. We have to avoid giving more salience to the semantics of a propositional variable over the others, and it is imperative to select the semantics of variables in a way such that they do not share characteristics that might escape our propositional grammar: for example, if the propositional variables were represented as circles that can be distinctly colored or not, it would be quite natural to assume that counting colored or uncolored circles could provide information, but this option is not considered in a theoretical design that assumes only propositional operators to describe rules. A related consideration is that we must also avoid introducing other regularities extraneous to the propositional formulation: if the images corresponding to all propositional variables are always shown in a straight line in the same order, salience effects might appear *even if* we avoid semantics that become more expressive thanks to the ordered nature of the represented variables (such as with descriptions of the form *the first and last elements are of the same size*).

#### Building adequate semantic representations for our logic

Taking these precautions into account, we choose to match each propositional variable with a particular image or figure, whose position in a square would be randomized (but avoiding superpositions). It is harder to decide exactly what would be the matching, but our final decision consists in matching each propositional variable with a set of two related Unicode characters (such as a triangle when the variable is 0, and a circle otherwise). See Fig. 4 for the exact representations. We take care to choose different types of characters for different variables: having *A*,*B* for *p*_1_ and *Y*,*Z* for *p*_5_ is out as a possibility, since it naturally introduces counting of the type ‘there is no more than 1 letter’ and the like. Of course, this process is not fail-safe, as there are countless possible semantics associations that could introduce extra-propositional grammar into the experiment. But we try to minimize the chance that this happens easily or naturally, and we use the written explanation stage as a way to catch these exceptions if they occur^5^.

Finally, to minimize possible salience effects from showing symbols that could have (despite our intentions to the contrary) different levels of conspicuousness, we randomize on a per-participant basis the assignment between pairs of symbols and propositional variables (but we do not randomize the assignment to the positive or negative value of a variable; the same Unicode characters are always positive in all randomizations, or always negative).

#### Ordering of positive and negative examples.

As mentioned before, in the learning stage we shuffle the order in which their positive and negative examples are shown, but always presenting all positive examples first. Also, the number of positive examples is smaller or equal to the number of negative examples for all concepts (see Table 1).

The purpose of placing the positive examples first and having less positive examples than negative ones is to bias the participant into thinking of the concept by its positive formulation, instead of possibly thinking of a rule that would describe the negative examples, and then negating that rule to obtain the positive one. This becomes important when we want to reason about the ease of learning of different operators: the default assumption is that participants that correctly select positive examples of the concept are thinking the positive rule, which differs in its operator from the negative rule (by the De Morgan laws).

## Results

### Hypothesis I

We asked whether the conjunction-disjunction bias (which is known to affect learning times in the case of a single explanation Bourne, 1970) also determines which features are used to describe a concept when two alternative explanations are consistent with the observed universe. In the first trial, the observed examples were consistent with *p*_1_ ∨ *p*_2_ and with *p*_3_ ∧ *p*_4_. As explained in “Experiment setup”, in the generalization stage we can determine if participants explained the concept using {*p*_1_,*p*_2_} or {*p*_3_,*p*_4_}. We found that 77 of the 100 participants attended to {*p*_3_,*p*_4_}, which corresponds to an explanation that uses a conjunction. 11 participants attended to {*p*_1_,*p*_2_} (corresponding to the use of a disjunction for the explanation), and 12 participants selected examples in the generalization stage inconsistent with both *p*_3_ ∧ *p*_4_ and *p*_1_ ∨ *p*_2_. To test the significance of this result, we performed a permutation test. Under the null hypothesis that participants randomly choose between explaining the concept using features {*p*_1_,*p*_2_} and explaining it using {*p*_3_,*p*_4_}, the probability that 77 of the 100 participants attend to {*p*_3_,*p*_4_} is *P* < 10^− 12^. Thus we conclude that the observed difference is significant.

Note that it is in principle possible that the participant learned the concept with a focus on negative examples (B’s in Fig. 3) instead of on positive examples (A’s in Fig. 3) (i.e. finding a correct explanation for the negative examples and then negating that rule to obtain an explanation for the positive examples).

As we mention in Section 2, we induced a bias to understand the concept in the appropriate way by first presenting the positive examples in the learning phase and by asking them to click on the positive ones in the training phase. We note, however, that 9 participants gave verbal explanations consistent with focusing on the negative examples. In this particular trial, a reverse interpretation is problematic since the negation of a conjunction corresponds to a disjunction, and the negation of the disjunction to a conjunction (i.e. *p* ∧ *q* is logically equivalent to $\lnot (\lnot p\lor \lnot q)$). Thus, a more comprehensive analysis should take into account participants’ verbal explanations in this trial. However, even considering the worst-case scenario in which these 9 participants were originally regarded as part of the ‘conjunction’ group and they are now considered part of the ‘disjunction’ group, the conjunction-disjunction bias is still significant (*P* < 10^− 7^). We therefore conclude that, in this framework where multiple explanations are possible depending on the attended features, there is a bias favoring conjunctive explanations over disjunctive explanations.

### Hypothesis II

**Fig. 9:**
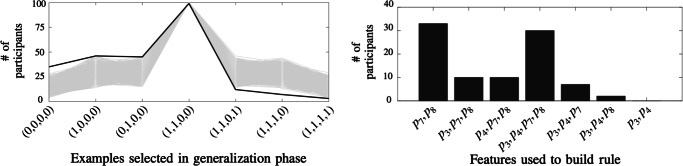
**(Left)** Number of participants (100 participants total) that, in the generalization stage of Trial 6, selected an element (possibly among others; the numbers add up to more than 100) with the elements written on the x-axis, indicating the values of the features {*p*_3_,*p*_4_,*p*_7_,*p*_8_} respectively. As multiple choices were possible, the sum for all choices adds up to a value greater than 100. In grey we show 100,000 simulations in which 100 agents randomly attend to one of the seven subset of features (see text). **(Right)** From the selected objects in the generalization phase we can infer which features participants used to build the rule for the concept (89 valid participants, see main text)

Most participants understood the concept in Trial 6 using the same features {*p*_7_,*p*_8_} used to describe the concept in Trial 5, even when the logical structure of the rule was exactly the same independently of attending to {*p*_7_,*p*_8_} or to {*p*_3_,*p*_4_}^6^. To show this, we study participants’ choices in the generalization stage of Trial 6 (see Fig. 9).

Suppose that a participant is thinking of the rule $\lnot p_{7} \land \lnot p_{8}$, thus they are only attending to features {*p*_7_,*p*_8_} while ignoring the features {*p*_3_,*p*_4_}. Since {*p*_3_,*p*_4_} are being ignored, the participant should mark those elements in which {*p*_7_,*p*_8_} agrees with the rule $\lnot p_{7} \land \lnot p_{8}$, irrespective of the values of {*p*_3_,*p*_4_}. That is, the participant should mark the elements with {*p*_3_,*p*_4_,*p*_7_,*p*_8_} equal to (0,0,**0,0**), (1,0,**0**,**0**), (0,1,**0**,**0**) and (1,1,**0**,**0**). These elements have {*p*_7_,*p*_8_} equal to (0,0) and ‘anything’ for {*p*_3_,*p*_4_}. On the other hand, if the participant is thinking of the rule $p_{3} \land \lnot p_{7} \land \lnot p_{8}$, then she is attending to {*p*_3_,*p*_7_,*p*_8_}, and she should mark (**1**,0,**0**,**0**) and (**1**,1,**0**,**0**).

In general, by studying which of the 7 examples shown in Fig. 9 (left) the participant selects in the generalization phase, we can deduce which features they were attending to (Fig. 9, right). For example, all participants should mark the example with {*p*_3_,*p*_4_,*p*_7_,*p*_8_} equal to (1,1,0,0), since it is consistent with all the logical rules irrespective of which features are used.

Indeed, as shown in Fig. 9 (left), all participants selected this example. Although in practice the participant can select any of the 7 examples in the generalization stage, we found that all but five participants respected the rules of coherence illustrated in the previous paragraph. These 5 participants were ‘one example away’ of respecting the rule, however, we leave them out of the feature stickiness analysis, but including them does not change our conclusions. We also excluded 6 participants that selected elements with no clear rationale in the previous trial, since they may not have used features {*p*_7_,*p*_8_}. However, including these participants (and assuming they did use {*p*_7_,*p*_8_} in the previous trial) does not significantly change the results. In total, these two exclusions leaves 89 participants for this analysis. The grey lines in Fig. 9 (left) show simulations of agents that randomly select one of the seven possible subsets of features, and then proceed to select the examples consistent with the logical rule using that features. Participants responses (black line) were biased towards explanations using {*p*_7_,*p*_8_}, as predicted by the feature-stickiness bias. This can also be seen in Fig. 9 (right), after inferring which features participants used to build the rule for the concept. In addition to being biased towards {*p*_7_,*p*_8_}, several participants explained the concept using all available features {*p*_3_,*p*_4_,*p*_7_,*p*_8_}. This shows that, in addition to the feature stickiness bias, when the number of features is relatively small, participants were also biased to describe the concept using all available features.

To quantify the feature stickiness bias, we assign a score to each participant according to the attended features in Trial 6 (deduced from the marked examples). The scores for the subsets {*p*_7_,*p*_8_}, {*p*_3_,*p*_7_,*p*_8_}, {*p*_4_,*p*_7_,*p*_8_}, {*p*_3_,*p*_4_,*p*_7_,*p*_8_}, {*p*_3_,*p*_4_,*p*_7_}, {*p*_3_,*p*_4_,*p*_8_} and {*p*_3_,*p*_4_} are 1, 2/3, 2/3, 1/2, 1/3, 1/3 and 0 respectively^7^. The average score for the 89 participants was 0.68 (*P* < 10^− 6^ in a permutation test with the null hypothesis of randomly attending to one of the seven subsets of features, which correspond to the grey lines in Fig. 9), indicating a significant effect of the feature stickness bias. Although the feature stickiness bias was significant for both groups independently (Group X: average score 0.62, *P* < 10^− 5^; Group Y: average score 0.74, *P* < 10^− 6^), we found that feature stickiness was higher in Group Y (two-sample t-test comparing the scores of the two groups shows *t* = 2.35, *P* < 0.05). The only difference between the groups is that Group Y had already (artificially) experienced feature stickiness between the previous Trials 3 and 4, so they have already identified it as an useful bias for the task. This suggests that the entire concept-learning sequence can be important when studying learning biases.

### Hypothesis III

**Fig. 10:**
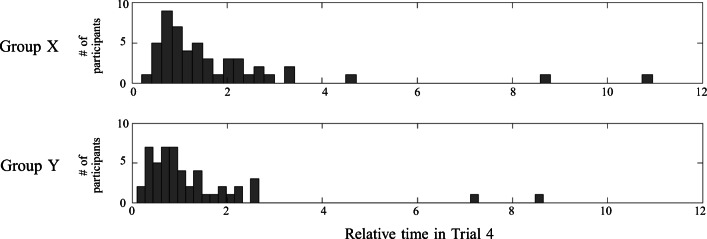
Relative time spent in Trial 4 by participants from the two groups, normalized by the time spent in Trial 5

This hypothesis regarded the behavioral advantage of the feature stickiness effect, which we tested by comparing learning *times* in Trial 4 for participants of Groups X versus Y (see Fig. 10). If the feature stickiness bias represents a behavioral advantage, Group Y should learn concept ${C^{4}_{1}}$ *faster* than Group X. To avoid confounds due to inter-individual differences in absolute learning time, for this analysis we normalize individual learning times with the time spent in Trial 5, which uses different features than the previous concepts and should not be affected by any obvious inter-trial relation with previous concepts^8^. Thus we compare between the two groups (X and Y) the time spent in Trial 4 divided the time expended in Trial 5. This gives one number for each participant, and we compare the lists of numbers of the two groups using a two-sample t-test. The differences in the learning times between the groups are not significant if we analyze the data of all participants as shown in Fig. 10 (two-sample t-test shows *t*_98_ = 1.26, *P* = 0.2; Cohen’s *d* = 0.25), but they are significant if we rule out from this analysis 5 outliers that spent more than 5 times in concept 4 than 5, or in concept 5 than 4 (*t*_98_ = 2.18, *P* < 0.05, Cohen’s *d* = 0.42)^9^.

### Hypothesis IV

The idea of this hypothesis is to test if participants prefer sticking to operators or sticking to features form one trial to the next. In this work we did not find conclusive evidence regarding this hypothesis. We suspect that the cause was an experimental setup that underestimated the strength of the bias favoring the ∧ operator over the ∨ operator. We found that 77 of the 100 participants explained Trial 1 using ∧, 11 explained it using ∨ and 12 selected elements in the generalization phase with no clear rationale. Of the 77 that used ∧, 64 also used ∧ in Trial 2, thus changing features but maintaining operator; and 7 of them used ∨, changing operator but maintaining features (the other 6 selected elements with no clear rationale). Of the 11 that used ∨, 10 used ∧ in Trial 2, changing operator but maintaining features; and 1 of them used ∨ in the second trial. We realize, however, that a change from using ∨ in the first concept to ∧ in the second one could not only be due to the effect of feature stickiness, but also simply to the stronger preference for ∧. Thus without a precise quantitative knowledge of the prior preference of ∧ over ∨, we cannot conclude about the effect of operator stickiness vs. feature stickiness. A future experiment could probe the existence of operator stickiness by having longer consecutive periods where feature reuse is not a useful bias and where only one logical operator remains useful for explaining a concept, before finally presenting a concept that can be explained via two different rules, each using different operators. Thus we leave for future work the task of studying the interaction between the feature stickiness bias and the precise structure of the logical rules being learnt.

### MDL bias

The MDL-bias hypothesis posits that concept-learning difficulty increases with its MDL (Feldman, 2000). In addition to their other roles, Trials 3 (group X and Y), 4, and 5 served to test this hypothesis in the new framework of multiple consistent explanations. In these trials, there were two possible explanations that were consistent with the shown data, one of much higher MDL than the other (15 vs. 3). For example, in the Group X of Trial 3, the short explanation was *p*_1_ ∧ *p*_2_, while the longer one was $((p_{3} \lor (p_{4} \lor p_{5}))\land (\lnot p_{3} \lor ((p_{4} \lor \lnot p_{5})\land (p_{5} \lor \lnot p_{4}))))$; the longer rule in other trials was always a substitution of features applied to this one (in order to keep the features disjoint between the two explanations). For these 3 trials, the responses of the 100 participants add to a total of 300 responses. From this total, 18 responses in the generalization phase did not choose objects consistent with any of the two explanations; 2 responses were consistent with the MDL 15 rule; and 280 responses were consistent with the MDL 3 rule. While this was expected by the experimental design (since we included a MDL 15 rule in those trials where we wanted to bias the participants into finding the other rule), we conclude that the MDL-bias hypothesis holds in this framework of multiple consistent explanations. Future work could explore in greater detail the relative difficulty of rules with slightly different MDL in this framework.

## Discussion

In this work, we design an experimental framework in which participants observe an incomplete set of examples, which are consistent with two alternative minimal descriptions depending on which features are observed. We illustrate several advantages of our method compared to separately presenting sets of examples consistent with only one minimal description at a time. First, we show that when a set of examples is consistent with a disjunction *and also* with a conjunction, participants are more likely to find the conjunction, in accordance with well-known previous results that show that the conjunction is learnt faster than the disjunction when presented separately (Bourne, 1970). Then, we show that when rules of significantly different MDL are consistent with the observations, almost all participants discover the simpler rules, consistent with previous result showing that, when rules of different MDL are tested separately, learning times are proportional to MDLs (Feldman, 2000). Finally, we show that when the logical structure of the minimal rules is independent of the selected features, participants are more likely to reuse the same features used to describe previous concepts, and preliminary results suggest that reusing features allows them to learn concepts faster than a control group that is not reusing features. To our knowledge this effect has not been previously characterized in the concept-learning literature, adding to the library of effects illustrating how human attention is biased towards features that are useful to describe the concepts (see Blair et al., 2009; Kruschke et al., 2000, 2005; Hoffman & Rehder 2010, among others).

Eye-tracking studies in categorization tasks have revealed that feature attention rapidly changes between trials depending on which features are relevant for classification in each trial (Blair et al., 2009), as well as depending on prior knowledge about feature relevance (Kim & Rehder, 2011). In Kruschke et al., (2005) it is found that eye movements confirmed that attention was learned in the basic learned inhibition paradigm, and in (Hoffman and Rehder, 2010) it is also found that eye movements revealed how an attention profile learned during a first phase of learning affected a second phase. Our experimental setup allows us to test an arguably simpler complementary hypothesis: everything else being equal, participants are biased to use the same features used in the past. Importantly, we were only able to test this hypothesis thanks to our framework, which allows us to present a set of examples consistent with two rules of exactly the same logical structure, but using different sets of features. Then, without using eye-tracking, we can recover which rule the participants learned, and thus which set of features they attended to. Since the two sets of features explain the examples using exactly the same logical structure, preferentially explaining the concept using one set of features over the other can only be due to a preference over the features themselves, and not a preference over alternative logical structures.

Although some of the hypothesis that we test are aligned with the well-known Einstellung effect which states that adopted solutions may hinder simpler ones when aiming at tackling novel problems, our experimental setting is different to the classical water jar test (the most commonly cited example of an Einstellung effect, where participants need to discover how to measure a certain amount of water using three jars with different and fixed capacity) (Luchins, 1942) in two senses. First, we do not drive the experiment to control and supervise the aspects that participants have to pay attention to. On the contrary, our focus is on the *choice* of the features that show to be useful for learning a concept with more than one rational explanation. Second, our experimental framework is consistent with the Language of Thought (LoT) hypothesis (Fodor, 1975), which states that the human capacity to describe concepts —and, more generally, of all elements of thought— builds on the use of a symbolic and combinatorial mental language and it is specifically conceived to handle expressions in propositional Logic (but expansible to other formal languages), which is the ground where the rational explanations can be formalized. Such approach enables us to treat the notion of *feature* in a very precise way.

We note that other frameworks besides LoT can be used for our experiment. For example, consider similarity-based classification rules (Juslin et al., 2003a, 2003b), where each feature is multiplied by a weight and the classification rule is a function of the sum of the weighted features, usually a linear function with a soft decision boundary (Juslin et al., 2003b). In this framework, the generalization phase would determine which of two possible decision boundaries was used by the participants (both consistent with the elements observed in the learning phase); and the feature-stickiness effect would be explained by the inertia of the weights’ values from one concept to the next. However, two obstacles in this framework makes us prefer the LoT framework for Boolean concept-learning tasks. First, although a linear classification rule can readily learn the conjunctions and disjunctions in our experiment, more complex classification rules would require nonlinear functions of the features (e.g. the exclusive-or (XOR)). For nonlinear boundaries, the values of the weights that accompany the features could be hard to interpret, since it might no longer be true that a higher weight means higher feature importance. In contrast, in the LoT framework complex classification rules are compositionally built to accommodate concepts of any complexity, and feature importance can always be modeled as the probability of including a feature in a formula, independently of its complexity. Second, unlike similarity-based rules, the LoT framework naturally explains how humans can built verbal explanations for the learned concepts. Indeed, almost all participants gave informal explanations of conjunctions and disjunctions in propositional logic after learning each concept (see the shared data online for the list of verbal explanations).

Another well-studied phenomenon related to our work is Kamin’s cue *blocking*, where the learning of a given stimulus B is *blocked* by the mere fact that it was preceded by a set of stimuli A that already pairs with the outcome. This shows that the subject learned that stimulus B was not useful, and hence disregards their attention to it in the upcoming events (Wagner, 1970; Mackintosh, 1975; Rescorla & Wagner, 1972). Studied in humans in Chapman and Robbins (1990), Arcediano et al., (1997), and Kruschke and Blair (2000) among others, our work differs from these approaches in that we never introduce a stage were a feature A is intentionally exposed in absence to B, in order to guide the attention of the participant.

We conjecture that most first-order determinants of subjective concept difficulty will also hold in a relative manner in our dual-concept setup, such as the MDL bias (for less extreme cases than evaluated in this work) (Feldman, 2003) and the transfer learning hierarchical structure bias (Tano et al., 2020). Importantly, our experimental setup also allows to directly test second-order subjective difficulty effects (e.g. concept A is learnt faster if presented jointly with concept B than with concept C), as well as second-order transfer learning effects (e.g. participants learn more rapidly concept C if they have first observed concept A coupled with B_1_, compared to A coupled with B_2_). We believe that a systematic study of concept-learning difficulty with two (or more) concepts presented at the same time in each trial may open a new window into the dynamics of human concept-learning mechanisms. For example, consider the study in Piantadosi et al., (2016), where participants gradually learn one concept while simultaneously selecting elements currently believed to belong to that concept. Here, the authors fit a Bayesian language model to participants’ choices in order to illustrate how the posterior probability of the different rules in the grammar varied across time, to approximate the order in which different rules are learned. In contrast, using our experimental setting we can directly estimate, in a model-free manner, the probability that each rule is learnt faster than another. One simply needs to jointly present (in an incomplete and mutually compatible way) a set of examples consistent with those two minimal rules, and then measure the fraction of participants that discover each rule.

Usually, concept-learning biases have been studied in an isolated manner: the participant observes examples indicated as inside or outside a *single* concept, and the experimenter evaluates its subjective difficulty for the participant. Although different methods have been used to present the concept to the participant (e.g. all elements at the same time (Tano et al., 2020; Kemp, 2012) or small sets of elements presented in series Piantadosi et al., 2016), to the best of our knowledge all previous category-learning studies have attempted to evaluate a single concept at a time. Here, we present a controlled logical setting to evaluate the relative difficulty of two concepts presented at the same time and under the same experimental conditions, and the framework could be generalized to more concepts straightforwardly.

## Appendix A: Exclusion criteria and data processing

We decided to collect data for up to 3 weeks or until we reached a total of 100 participants. Via restrictions on the platform where the experiment was conducted, participants that took more than 4 hours or who did not complete all the trials were automatically excluded from the analysis. We were also prepared to exclude afterward the results from those participants whose verbal explanations denoted the use of external aids or methods outside the scope of the paper, such as using external help or taking screenshots of the concept, but there were no clear-cut cases of that behaviour (*N* = 0).

Additionally, while our preregistered exclusion criteria did not encompass the potential cases of written explanations that were legitimate but indicative of use of rules extraneous to propositional logic or to our semantic framework, in the end we did not detect any of these cases. This encouraging result is weakly indicative of the usefulness of our careful considerations for building adequate semantic representations, as mentioned in “Notes on the experiment design”. For the comprehensive written explanations of the participants, we refer the reader to the uploaded raw data at https://osf.io/gtuwp/.

Balanced division into the two groups was handled via the psiTurk library, which decides the group a new worker will be assigned to, based on the current number of completed experiments in each group.

We ignored individual trails from participants that in the generalization stage chose a generalization inconsistent with any valid explanation (but this did not provoke the exclusion of other independent trials by the same participant). See “Results” for details.

## Appendix B: Pilot

This experiment is informed by a previous pilot with 22 participants, which we executed in order to have some validation for our expected effects before making the preregistration. This pilot used more complex pairs of concepts, with a longer minimum description length for the two corresponding rules, and where using both ∧ and ∨ in the same rule was often necessary. Originally, we expected a naturally arising separation into different groups, depending on the features of explanation found for the first trial. However, we encountered a very strong preference for explanations using solely ∧, and this prompted various changes in the final design of the experiment that was preregistered in the OSF version.

More precisely, in our first trial in that pilot, 81% (*N* = 18) of the workers explained the (incomplete) concept as a conjunction of three variables, while only 9% (*N* = 2) explained it as a disjunction of two. This happened even though we had made the ∧ explanation longer with the intention to compensate for the relative ease of ∧ with respect to ∨ (so as to avoid getting a statistically inadequate number of participants self-selecting to the ∨ case). This result goes in line with known work about the relative hardness of learning concepts with the ∨ operator (Bourne, 1970). In our framework of more than one plausible rule, a possible explanation to this population disparity could be that, when looking for common characteristics, it is natural to search first for individual features that always appear. Another explanation could be that, in a universe with low number of features, repetition of many of them becomes very salient, and thus the relation between hardness and number of conjunctions is not necessarily monotonic. In any case, this result was not part of the preregistration, so it is presented here only as an indication of an interesting effect to study.

## Appendix C: Technical results

Let us fix a non-empty set of propositional variables PROP. A valuation is formally defined as a function ${v:\textsc {PROP}\rightarrow \{0,1\}}$ that determines the truth value of the propositional variables. A valuation can be extended in the standard way to preserve the usual semantics of Boolean operators and thus to determine the truth value of propositional formulas (which we call ‘rules’ in the context of describing concepts). We say that a valuation *v* satisfies a formula *φ* if *v*(*φ*) = 1. We say that a formula *φ* is a contingency if there exist a valuation *v*_*t*_ that satisfies it and a valuation *v*_*f*_ that does not.

Given a propositional formula *φ*, we define VAR*φ* as the set of variables that appear in it. For example, if $\varphi _{e} = p_{1} \lor (p_{2} \land \lnot p_{2})$, then VAR*φ*_*e*_ = {*p*_1_,*p*_2_}.

We say that a formula *φ* is variable-minimal if there is no other formula *ψ* such that the truth values of *φ* and *ψ* coincide over all valuations and $\textup {VAR}{\psi } \subsetneq \textup {VAR}{\varphi }$. For example, the previous *φ*_*e*_ is not variable-minimal, since it is equivalent to *ψ* = *p*_1_, which uses one less propositional variable.

We begin by proving a very basic lemma for illustrative purposes.

### **Lemma 1**

Let *φ*_1_ and *φ*_2_ be two contingencies such that VAR*φ*_1_ ∩VAR*φ*_2_ = *∅*.

Then there exists a valuation *v*_*i**n*_ such that *v*_*i**n*_ satisfies both *φ*_1_ and *φ*_2_, and a valuation *v*_*o**u**t*_ that satisfies neither *φ*_1_ nor *φ*_2_.

In other words, the lemma says that when we have two non-trivial concepts concerning non-overlapping sets of features, then there is at least one (positive) example that satisfies both concepts simultaneously and at least one (negative) example that satisfies none of them.

### *Proof*

Whether a valuation satisfies or not a formula *φ* depends only on how it evaluates propositional variables on VAR*φ*. Since VAR*φ*_1_ ∩VAR*φ*_2_ = *∅* and both formula are satisfiable via some *v*_1_ and *v*_2_ respectively, we can construct a valuation *v*_*i**n*_ by joining the values of *v*_1_,*v*_2_ on the (disjoint) sets of variables of each formula: *v*_*i**n*_(*p*) = *v*_1_(*p*) if *p* ∈VAR*φ*_1_, *v*_*i**n*_(*p*) = *v*_2_(*p*) if *p* ∈VAR*φ*_2_, and *v*_*i**n*_(*p*) = 0 otherwise.

Similarly, since *φ*_1_,*φ*_2_ are not contingencies, there exist valuations $\bar {v}_{1}$ and $\bar {v}_{2}$ that do not satisfy *φ*_1_ and *φ*_2_ respectively. We use these valuations as before to construct a valuation *v*_*o**u**t*_ that does not satisfy *φ*_1_ nor *φ*_2_, as we wanted. □

### **Lemma 2**

If *φ* is a variable-minimal contingency, and *p* ∈VAR*φ*, then there exists a valuation *v* such that *v* satisfies *φ* but $\tilde {v}$ does not, where $\tilde {v}$ is the single valuation that coincides with *v* except on *p*.

### *Proof*

By way of contradiction, assume the conclusion does not hold: that for any valuation, its satisfaction of *φ* is independent of its value on *p*. In this case, necessarily {*p*}≠VAR*φ*, or otherwise *φ* would not be a contingency (as it would always be true or always false).

Now consider *V*_*φ*_ the (non-empty) set of valuations that satisfy *φ*, and consider $V^{-p}_{\varphi }$ its restriction to VAR*φ*∖{*p*}. From $V^{-p}_{\varphi }$ we can construct, in a standard way via truth tables, a formula $\tilde {\varphi }$ with $\textup {VAR}{\tilde {\varphi }} = \textup {VAR}{\varphi } \backslash \{p\}$ such that a valuation *v* satisfies $\tilde {\varphi }$ if and only if $v|_{\textup {VAR}{\tilde {\varphi }}} \in V^{-p}_{\varphi }$. Since by assumption the value of *p* does not matter for *φ*, we have by construction that *φ* is equivalent to $\tilde {\varphi }$, but $\textup {VAR}{\tilde {\varphi }} \subsetneq \textup {VAR}{\varphi }$, which contradicts the variable-minimality of *φ*. □

The following theorem shows the general theoretical correctness of our experimental setup. It says that if we show as positive examples the full intersection of two non-trivial concepts whose minimal descriptions contain no features in common, and show as negative examples the complement of the union of both concepts, any rule used to explain the seen (incomplete) concept must use a superset of the variables used to minimally describe one of these concepts. Otherwise, the chosen rule would be incompatible with the known data.

### **Theorem 3**

Let *φ*_1_ and *φ*_2_ be two variable-minimal contingencies such that VAR*φ*_1_ ∩VAR*φ*_2_ = *∅*. Let *ψ* be a formula such that VAR*ψ* ∩VAR*φ*_1_≠VAR*φ*_1_ and such that VAR*ψ* ∩VAR*φ*_2_≠VAR*φ*_2_. Furthermore, assume that for all valuations *v* that satisfy *φ*_1_ ∧ *φ*_2_, *v* also satisfies *ψ*. Then there exist two valuations *v*_*i**n*_,*v*_*o**u**t*_ such that:

1. *v*_*i**n*_ satisfies *φ*_1_ ∧ *φ*_2_
2. *v*_*o**u**t*_ does not satisfy *φ*_1_ ∨ *φ*_2_
3. *v*_*i**n*_ and *v*_*o**u**t*_ both satisfy *ψ*.

### *Proof*

From the hypotheses we know that there is a variable *p*_1_ ∈VAR*φ*_1_∖VAR*ψ* and a variable *p*_2_ ∈VAR*φ*_2_∖VAR*ψ*. Since *φ*_1_,*φ*_2_ are variable-minimal contingencies, from Lemma 2 we have that there exist valuations *v*_1_ and *v*_2_ such that they satisfy *φ*_1_ and *φ*_2_ respectively, but where $\tilde {v}_{1}$ and $\tilde {v}_{2}$ do not, with $\tilde {v}_{1}$ and $\tilde {v}_{2}$ being the valuations that coincide with *v*_1_ and *v*_2_ save on *p*_1_ and *p*_2_ respectively. Using that VAR*φ*_1_ ∩VAR*φ*_2_ = *∅*, we can construct from *v*_1_ and *v*_2_ (as we did in the proof of Lemma 1) a valuation *v*_*i**n*_ such that *v*_*i**n*_ satifies both *φ*_1_ and *φ*_2_, and also such that *v*_*o**u**t*_ does not satisfy neither of them, where we take *v*_*o**u**t*_ to coincide with *v*_*i**n*_ save on *p*_1_ and on *p*_2_. From the hypothesis, necessarily *v*_*i**n*_ satisfies *ψ*. However, since {*p*_1_,*p*_2_}∩VAR*ψ* = *∅*, the value over *p*_1_ or *p*_2_ does not matter for the satisfaction of *ψ*, and thus *v*_*o**u**t*_ also satisfies *ψ*, as we wanted to see. □

Note that the statement of Theorem 3 can be generalized to any number of non-trivial rules $\varphi _{1}, \dots , \varphi _{n}$ such that VAR*φ*_*i*_ ∩VAR*φ*_*j*_ = *∅* for all *i*≠*j*, and with *ψ* such that VAR*ψ* ∩VAR*φ*_*i*_≠VAR*φ*_*i*_ for all *i*. This means that we can test concept learning under any multiplicity of possible explanations, as long as the underlying propositional universe is large enough and the rules are chosen adequately.

## Footnotes

1 With the exception of Trial 6, where one element is reshown in order to better test Hypothesis II. See “Experiment trials”.

2 Note that the features {*p*_5_,*p*_6_} that were used in Trial 4 also appear in the MDL15 formula of Trial 5. However, we hypothesized that the extreme complexity of the MDL15 explanation overwheights the possible feature stickiness effect from Trial 4 to 5. Indeed, we found that none of the participants used the MDL15 formula in Trial 5.

3 The Trial 6 is an exception, and has an element that is consistent with both rules.

4 The rules that are actually ‘learnable’ use exactly 2 propositional variables.

5 In the end, they did not occur. See Appendix ??.

6 As expected by our experiment design, 94 of the 100 participants understood the concept in Trial 5 using features {*p*_7_,*p*_8_} (6 selected features with no clear rationale). Using features {*p*_7_,*p*_8_} is indeed the only plausible way to learn the concept, given the high complexity of the alternative MDL15 formula.

7 Part (d) of the Analysis Plan section in our preregistration had a mistake in the use of features names: the learnable concept corresponding to the fifth trial uses *p*_7_ and *p*_8_, not *p*_3_ and *p*_4_ as erroneously written in that part; compare with the section on Study design, which matches Table 1.

8 Indeed, Trial 5 was pre-registered as a ‘normalizer’ trial.

9 The ANOVA proposed in the pre-registration also did not reveal significant differences in learning times. For simplicity in the analysis of the outliers, we replaced here the ANOVA for a simple t-test between the normalized learning times of the two groups.

## References

[CR1] Arcediano F, Matute H, Miller RR (1997). Blocking of pavlovian conditioning in humans. Learning and Motivation.

[CR2] Ashby FG, Maddox WT (2005). Human category learning. Annual Review of Psychology.

[CR3] Ashby FG, Maddox WT (2011). Human Category learning 2.0. Annals of the New York Academy of Sciences.

[CR4] Blair M, Homa D (2003). As easy to memorize as they are to classify: The 5–4 categories and the category advantage. Memory & Cognition.

[CR5] Blair MR, Watson MR, Walshe RC, Maj F (2009). Extremely selective attention: Eye-tracking studies of the dynamic allocation of attention to stimulus features in categorization. Journal of Experimental Psychology: Learning, Memory, and Cognition.

[CR6] Bourne LE (1970). Knowing and using concepts. Psychological Review.

[CR7] Buhrmester M, Kwang T, Gosling SD (2011). Amazon’s Mechanical Turk: A new source of inexpensive, yet high-quality, data?. Perspectives on Psychological Science.

[CR8] Chapman GB, Robbins SJ (1990). Cue interaction in human contingency judgment. Memory & Cognition.

[CR9] Cohen, H., & Lefebvre, C. (2005). *Handbook of categorization in cognitive science*. Elsevier.

[CR10] Crump MJ, McDonnell JV, Gureckis TM (2013). Evaluating Amazon’s Mechanical Turk as a tool for experimental behavioral research. PLOS ONE.

[CR11] Feldman J (2000). Minimization of Boolean complexity in human concept learning. Nature.

[CR12] Feldman J (2003). The simplicity principle in human concept learning. Current directions in psychological science.

[CR13] Fodor, J. A. (1975). *The language of thought*, vol. 5. Harvard University Press.

[CR14] Grünwald, P.D., & Grunwald, A. (2007). *The minimum description length principle*. MIT Press.

[CR15] Hoffman AB, Rehder B (2010). The costs of supervised classification: The effect of learning task on conceptual flexibility. Journal of Experimental Psychology: General.

[CR16] Juslin P, Jones S, Olsson H, Winman A (2003). Cue abstraction and exemplar memory in categorization. Journal of Experimental Psychology: Learning, Memory, and Cognition.

[CR17] Juslin P, Olsson H, Olsson A-C (2003). Exemplar effects in categorization and multiple-cue judgment. Journal of Experimental Psychology: General.

[CR18] Kemp C (2012). Exploring the conceptual universe. Psychological Review.

[CR19] Kim S, Rehder B (2011). How prior knowledge affects selective attention during category learning: an eyetracking study. Memory & Cognition.

[CR20] Kruschke JK, Blair NJ (2000). Blocking and backward blocking involve learned inattention. Psychonomic Bulletin & Review.

[CR21] Kruschke JK, Kappenman ES, Hetrick WP (2005). Eye gaze and individual differences consistent with learned attention in associative blocking and highlighting. Journal of Experimental Psychology: Learning, Memory, and Cognition.

[CR22] Lewandowsky S (2011). Working memory capacity and categorization: individual differences and modeling. Journal of Experimental Psychology: Learning, Memory, and Cognition.

[CR23] Luchins, A. S. (1942). Mechanization in problem solving: The effect of einstellung, (Vol. 54.

[CR24] Mackintosh NJ (1975). A theory of attention: Variations in the associability of stimuli with reinforcement. Psychological Review.

[CR25] Maddox WT, Ashby FG (1993). Comparing decision bound and exemplar models of categorization. Perception & Psychophysics.

[CR26] Minda JP, Smith JD (2001). Prototypes in category learning: the effects of category size, category structure, and stimulus complexity. Journal of Experimental Psychology: Learning, Memory, and Cognition.

[CR27] Nosofsky RM, Gluck MA, Palmeri TJ, McKinley SC, Glauthier P (1994). Comparing modes of rule-based classification learning: a replication and extension of shepard, hovland, and jenkins (1961). Memory & cognition.

[CR28] Nosofsky RM, Palmeri TJ, McKinley SC (1994). Rule-plus-exception model of classification learning. Psychological Review.

[CR29] Piantadosi ST, Tenenbaum JB, Goodman ND (2016). The logical primitives of thought: Empirical foundations for compositional cognitive models. Psychological review.

[CR30] Rehder B, Hoffman AB (2005). Eyetracking and selective attention in category learning. Cognitive Psychology.

[CR31] Rescorla, R. A., & Wagner, A. R. (1972). A theory of Pavlovian conditioning: Variations on the effectiveness of reinforcement and non-reinforcement. In A. H. Black, & W. F. Prokasy (Eds.) *Classical conditioning II: Current research and theory* (pp. 64–99). New York: Appleton-Century-Crofts.

[CR32] Schyns PG, Goldstone RL, Thibaut J-P (1998). The development of features in object concepts. Behavioral and Brain Sciences.

[CR33] Shepard RN, Hovland CI, Jenkins HM (1961). Learning and memorization of classifications. Psychological Monographs: General and Applied.

[CR34] Stewart N, Ungemach C, Harris AJ, Bartels DM, Newell BR, Paolacci G, Chandler J (2015). The average laboratory samples a population of 7,300 Amazon Mechanical Turk workers. Judgment and Decision Making.

[CR35] Tano P, Romano S, Sigman M, Salles A, Figueira S (2020). Towards a more flexible language of thought: Bayesian grammar updates after each concept exposure. Phys. Rev. E.

[CR36] Tenenbaum JB, Kemp C, Griffiths TL, Goodman ND (2011). How to grow a mind: Statistics, structure, and abstraction. Science.

[CR37] Wagner, A. R. (1970). Stimulus selection and a ”modified continuity theoryrdquo. In *Psychology of learning and motivation*, (Vol. 3 pp. 1–41): Elsevier.

